# Profiles of Behavioral Self-Regulation and Appetitive Traits in Preschool Children: Associations With BMI and Food Parenting Practices

**DOI:** 10.3389/fnut.2022.796580

**Published:** 2022-03-04

**Authors:** Lori A. Francis, Brandi Y. Rollins, Kathleen L. Keller, Robert L. Nix, Jennifer S. Savage

**Affiliations:** ^1^Department of Biobehavioral Health, The Pennsylvania State University, University Park, PA, United States; ^2^Department of Nutrition Sciences, The Pennsylvania State University, University Park, PA, United States; ^3^Department of Human Development and Family Studies, University of Wisconsin-Madison, Madison, WI, United States

**Keywords:** self-regulation, appetitive traits, food approach, food avoidance, childhood obesity, food parenting practices, latent profile analysis

## Abstract

Appetitive traits that contribute to appetite self-regulation have been shown to relate to non-food-related regulation in general domains of child development. Latent profile analysis (LPA) was used to identify typologies of preschool children's behavioral self-regulation (BSR) and appetitive traits related to appetite self-regulation (ASR), and we examined their relation with children's BMIz and food parenting practices. Participants included 720 children and their parents (90% mothers), drawn from the baseline assessment of a childhood obesity preventive intervention. BSR measures included teacher reports of children's inhibitory control, impulsivity and attentional focusing, as well as an observed measure of inhibitory control. ASR was assessed using parents' reports of children's appetitive traits related to food avoidance (*e.g.*, satiety responsiveness, slowness in eating) and food approach (*e.g.*, enjoyment of food, food responsiveness). Children's body mass index z-score (BMIz) was calculated from measured height and weight. Parents' BMI and food parenting practices were also measured. Four profiles were identified that characterized children with dysregulated behavior, higher food approach and lower food avoidance (16%), dysregulated behavior but lower food approach and higher food avoidance (33%), regulated behavior but highest food approach and lowest food avoidance (16%), and highly-regulated behavior, lowest food approach and highest food avoidance (35%). Children's BMIz was highest in the profile consisting of children with dysregulated behavior, higher food approach and lower food avoidance. BMI was similar in the profile with children with regulated behavior but highest food approach and lowest food avoidance; children in this profile also had parents who reported the highest levels of controlling food parenting practices, and the lowest levels of parental modeling of healthy eating. Compared to all other profiles, children in the profile characterized by highly-regulated behavior, lowest food approach and highest food avoidance had the lowest BMIz and had parents who reported food parenting practices characterized by the highest levels of child control in feeding and the lowest levels of pressure to eat. These findings provide evidence of differing patterns of relations between self-regulation across behavioral and eating domains, and children's obesity risk may vary based on these different patterns.

## Introduction

Childhood obesity is a major public health challenge in the U.S. and across the world (1). Approximately 12% of U.S. children ages 2–5 were classified with obesity between 2013 and 2016 (2). Children from low-income households are more likely to have obesity, compared to children from middle- or high-income households (3, 4). Deficits in self-regulatory capacity, the ability to control an impulse or behavior, in general domains of development have been implicated in the development of obesity (5–9). General self-regulation is a broad term that is used to describe a number of behavioral, emotional, and cognitive processes related to one's ability to plan and structure behaviors, focus attention, and inhibit impulses to pursue long-term goals (10). Self-regulatory behavior can be measured across multiple domains of development (e.g., biological, behavioral, emotional, eating), and are essential for biobehavioral health and successful development throughout childhood (11). Evidence linking self-regulation to childhood obesity suggests that greater deficits in children's early self-regulatory capacity (~age 3 or 4 years) may be linked to rapid weight gain and obesity through adolescence, (6, 8) and into adulthood (12).

As described by Nigg (13), self-regulation includes both top-down and bottom-up processes that co-act. Bottom-up processes are automatic, and require little effort to enact, whereas top-down processes are deliberate, goal-based and require cognitive effort and control. Executive function processes, which represent neurocognitive processes related to problem solving, planning, reasoning and goal-directed behaviors (14), are top-down components of self-regulation that have been implicated in the development of obesity (15, 16). Although executive function processes related to behavioral inhibition (*e.g.*, inhibitory control and impulsivity) have been the most widely-researched processes in studies linking executive function to obesity (15), the exact mechanisms through which executive function is linked to the development of obesity in childhood are not yet fully understood. However, inhibitory control and impulsivity are thought to be implicated in the etiology of obesity, in part, through their influence on children's appetitive traits related to appetite self-regulation.

Appetite self-regulation, as described by Russell and Russell (17), refers to neurocognitive, social and biobehavioral processes or skills involved in an individual's ability to regulate energy intake. Appetitive traits include several domains of eating behaviors, most of which are bottom-up processes that contribute to appetite self-regulation, although some include an interplay of top-down and bottom-up processes (e.g., satiety responsiveness) (18). Appetitive traits have been conceptualized as traits that may explain individuals' differential susceptibility to food, which may confer differential levels of risk for or resilience from obesity (19). Several appetitive traits are conceptualized as a set of eating behaviors that indicate children's tendency toward food approach (i.e., responsiveness to food stimuli, such as the presence of food) and food avoidance (*e.g.*, responsiveness to cues that signal fullness). These traits have been associated with young children's appetite self-regulation and weight status (20–27), and show a small to moderate degree of stability from early (ages 3–5) to late childhood (ages 9–11) (28, 29).

Food approach behavior is described as a movement toward or desire for food, which includes traits such as a preoccupation with food, and eating in response to external or emotional cues. Food avoidance behavior is described as a movement away from food, and includes traits such as picky eating/food fussiness, a slow eating rate, and satiety responsiveness, which is a sensitivity to cues that signal fullness (22, 30). These appetitive traits have consistently been shown to be related to preschool children's obesity risk (31–33), with a higher risk in children who exhibit greater tendencies toward food approach behavior and lower risk in children with greater tendencies toward food avoidance behavior. Findings from multiple studies provide evidence for an interplay between appetitive behaviors and neurocognitive and behavioral systems related to general self-regulation, although there may be underlying processes that are domain specific (18). In a study of 187 low-income, Hispanic preschool children, Hughes and colleagues (23) found that general self-regulation was associated with children's satiety responsiveness, but not with objectively-measured eating regulation (e.g., eating in the absence of hunger) or BMIz; only eating-related regulation measures were associated with child BMIz. In a study with a predominantly white, middle- and upper-income sample of children ages 3–6 years, Giuliani and Kelly (34) assessed children's delay of gratification on a food-based task, which measured whether children chose to wait for a larger snack portion over receiving an immediate, smaller snack portion. The authors found that children's inability to delay gratification (choosing the immediate, smaller portion) was related to greater, objectively-measured eating in the absence of hunger, but it was not related to tasks measuring general, Non-food self-regulation (attentional and inhibitory control) or BMIz. Neither eating- or Non-eating-related regulation was related to child BMIz in the Guiliani et al. study. Additional studies are needed that elucidate the ways in which self-regulation may be linked across domains (*i.e.*, food and Non-food related), and the extent to which various combinations of regulation-related individual, family and household factors increase risk for or confer protection from obesity in children.

One such factor may be coercive food parenting practices, including Non-responsive, controlling attempts to alter children's food intake. Such food parenting practices may undermine children's ability to regulate their intake, and influence the development of obesity in young children (35). Conversely, child-focused, responsive food parenting practices have been shown to promote healthy eating and weight outcomes in children (35). The combined effects of appetitive behaviors, general self-regulation and food parenting practices on children's obesity risk have been examined in several studies (36–41). Although the findings are somewhat mixed, there is evidence to suggest that the effects of coercive food parenting practices on children's obesity risk appear to be exacerbated in children who exhibit appetitive behaviors associated with deficits in eating regulation. Rollins et al. (37) found that the effects of maternal controlling food parenting practices on 5-year-old girls' eating regulation and BMI was most pronounced in girls with low inhibitory control. There are also findings that show that food parenting practices may moderate the association between children's appetitive behaviors and obesity risk. Vollmer et al. (42) interviewed 150 racially- and socioeconomically diverse fathers of children ages 3–5 years and found that the inverse relation between satiety responsiveness and preschool children's BMIz was only significant in children with fathers who used coercive food parenting practices.

Given the multifactorial nature of obesity, there is a confluence of factors across multiple levels of influence that impact children's risk for obesity. Russell and Russell (43) highlight the need for a biopsychosocial approach to research on the development of obesity in children, and call for integrated models that examine links between individual factors across multiple domains of development (e.g., children's appetitive traits and behavioral self-regulation) and parent-related (e.g., food parenting practices) factors, and their interactive roles on children's risk for obesity. This approach formed the basis of the conceptual framework for the present study, along with the dual processing model that conceptualizes self-regulation as involving interplay between top-down regulatory processes (i.e., inhibitory control) and bottom-up regulatory processes (i.e., food approach/avoidance) (18). The objectives of the current study were to use a person-centered approach, latent profile analysis, to explore typologies of preschool children's behavioral self-regulation (BSR), food approach and food avoidance, and their relation with children's BMIz, parents' BMI and parents' food parenting practices. We hypothesized that (a) distinctive profiles of BSR and appetitive traits would be identified; (b) profiles characterized by high BSR, low food approach and high food avoidance would be associated with lower child BMIz and more responsive food parenting practices; and (c) profiles characterized by low BSR, high food approach and low food avoidance would be associated with higher child BMIz and more coercive food parenting practices. To account for the potential genetic and environmental influence of parental weight status on study outcomes, we also examined relations with parental BMI. We hypothesized that higher parental BMI would predict children's membership in the most dysregulated profiles, characterized by combinations of weaker top-down regulatory control (lower inhibitory control and attentional focusing and higher impulsivity), high food approach and low food avoidance.

## Materials and Methods

### Participants

All data were drawn from the Healthy Bodies Project, a 28-week childhood obesity preventive intervention conducted in center-based childcare programs in Central and Southcentral Pennsylvania; 57% of participating centers served predominantly low-income families. Only data collected at baseline (before the intervention began) between 2017 and 2020 were utilized in the current study. To be included in analyses for the current study, surveys from teachers and parents were required; out of the 1397 eligible children, 720 met those criteria. Mothers (90%) represented the majority of parents who completed the parent survey; 9% of parents were fathers, and the remaining respondents were stepmothers and related caregivers. All procedures were approved by the Pennsylvania State University Institutional Review Board.

### Measures

#### Behavioral Self-Regulation (BSR)

Children's behaviors related to self-regulation were assessed using the Children's Behavior Questionnaire–Teacher's Short Form (CBQ-TSF) (44) and the Walk a Line Slowly behavioral task (45, 46). On the CBQ-TSF, teachers reported each child's level of inhibitory control, impulsivity, and attention focusing. Inhibitory control (reported by the teacher) refers to the capacity to plan actions and inhibit inappropriate responses (e.g., “Can easily stop an activity when s/he is told ‘no”'; α = 0.85). Impulsivity refers to the speed of initiating a response, or acting without thinking (e.g., “Often rushes into new situations”; α = 0.78). Attentional focusing refers to the ability to maintain attention and focus on a task (e.g., “When drawing or coloring in a book, shows strong concentration”; α = 0.87). Each subscale consisted of 6-items; response options were on a 7-point scale ranging from 1 (extremely untrue of your child) to 7 (extremely true of your child). Teachers are also given the option to select “Not applicable (N/A).” An adapted version of the Walk a Line Slowly behavioral task, or “Turtle Race,” provided a measure of inhibitory control (observed in the classroom). In the original version of this task, each child is asked to slowly walk down a “path” consisting of a 2.5-inch x 12-foot strip of colorful tape. Due to concerns about classroom space constraints, a 6-foot line of green-colored tape was used in the current study. As in the original task, a baseline trial was followed by two trials in which children were asked to walk down the line as slowly as they can and then even slower; the length of time (in seconds) it took for the child to walk the line was recorded for each trial, and the two Non-baseline trials were averaged to comprise an observed measure of inhibitory control. Both the teacher-reported and classroom observed measures of inhibitory control were moderately related in this sample (*r* = 0.25, *p* < 0.001).

#### Appetitive Traits

Children's appetitive traits were measured using parent reports on the Child Eating Behavior Questionnaire (47). Reliability estimates have been found to be satisfactory with low-income samples of parents of preschoolers (48–51). For the purposes of this study, we included two subscales that indicate *food avoidance behaviors* (movement away from food), and three subscales that indicate *food approach behaviors* (movement toward food) (22). These subscales have been widely reported in the literature to be associated with young children's weight status, objectively-measured eating self-regulation, and food parenting practices, among other relevant outcomes (19, 30, 33, 51, 52). From the *food avoidance* domain, we included the satiety responsiveness subscale, referring to the ability to stop eating in response to satiety cues (e.g., “My child leaves food on his/her plate at the end of a meal”; α = 0.69) and slowness of eating subscale (e.g., “My child takes more than 30 min to finish a meal”; α = 0.72). From the *food approach* domain, we included the enjoyment of food subscale (e.g., “My child loves food”; α = 0.85), the food responsiveness subscale (e.g., “Even if my child is full up s/he finds room to eat his/her favorite food”; α = 0.74), and the emotional overeating subscale (e.g., “My child eats more when worried”; α = 0.76). Each subscale consisted of 3- to 5-items, and response options were on a 5-point scale ranging from 1 (never) to 5 (always). For the purposes of this study, we conceptualize food avoidance and food approach behaviors as processes that contribute to children's appetite self-regulation. For the purposes of this study, children higher on food avoidance and lower on food approach are characterized as higher in appetite self-regulation, and children higher in food approach and lower on food avoidance are characterized as lower in appetite self-regulation.

#### Sociodemographics

In the parent survey, parents reported on their child's age, sex (0 = male, 1 = female), and race (recoded as 0 = white Non-Hispanic, 1 = child of color). Parents also self-reported on their age, education levels (recoded as 0 =< college; 1 = completed college or more), and household income (1 = “<$20,000”, 2 = “$20,000 to 34,999”, 3 = “$35,000 to 49,999”, 4 = “$50,000 to 75,000”, 5 = over $75,000).

#### Anthropometrics

Children's height and weight were measured in triplicate using standardized procedures by trained research assistants in the preschool setting. Height was measured to the nearest 0.1 cm and weight was measured to the nearest 0.1 kg; shoes and heavy clothes were removed. Height and weight were used to calculate age- and sex-specific body mass index (BMI; kg/m^2^), and BMI percentiles and z-scores based on standardized reference criteria recommended by the Centers for Disease Control and Prevention (53). Weight status classifications included: Non-overweight (BMI < 85th percentile), overweight (BMI ≥ 85th percentile), and obesity (≥95th BMI percentile). Parents were asked to self-report their current height (inches) and weight (pounds) in the online parent survey. These data were used to compute parents' BMI scores (weight[kg]/height[m^2^]).

#### Food Parenting Practices

Parents reported their food parenting practices on the Comprehensive Feeding Practices Questionnaire (54), a 49-item measure of parenting in the feeding domain. For the purposes of this paper, we included subscales measuring feeding constructs that have been shown to support or undermine children's self-regulation (35, 55, 56). Responsive feeding subscales included the child control subscale (e.g., “Do you let your child eat whatever s/he wants?”; α = 0.67), which measures autonomy-granting in feeding or the degree to which parents allow children to control their own eating; modeling (e.g., “I model healthy eating for my child by eating healthy foods myself”; α = 0.83); and monitoring (e.g., “How much do you keep track of sweets?”; α = 0.88). Coercive feeding subscales included food as reward (e.g., “I offer my child his/her favorite foods in exchange for good behavior”; α = 0.69); emotion regulation (e.g., “Do you give this child something to eat or drink if s/he is upset even if you think s/he is not hungry?”; α = 0.80); pressure to eat (e.g., “My child should always eat all of the food on his/her plate”; α = 0.66); restriction for health (e.g., “I have to be sure that my child does not eat too many sweets”; α = 0.74); and restriction for weight control (e.g., “I restrict the food my child eats that might make him/her fat”; α = 0.80). Internal consistency estimates are similar to those reported by others, (54, 57, 58) including lower estimates on the food as reward and child control subscales. Each subscale consisted of 3 to 8 items, and response options ranged from 1 (never/disagree) to 5 (always/agree).

### Statistical Analysis

To address the study research questions, first, we examined bivariate relations among children's BSR, food approach and food avoidance traits. Second, we utilized latent profile analyses to identify profiles of BSR and these appetitive traits that capture the interactive relation between these variables. Third, we investigated whether individual differences in child BMI, and parent-reported food parenting practices and BMI were related to membership in the identified profiles.

Pearson correlations were computed to examine bivariate relations among the measures of behavioral self-regulation and appetite self-regulation. To accommodate the small amount of missing data (1% of data points affecting 30 participants), 25 multiple imputations were performed in SAS 9.4 (SAS Institute Inc., Cary, NC, USA) (59). All study variables were included in the imputation model.

Latent profile analysis (LPA) was conducted in Mplus 8.0 (Muthen & Muthen, Los Angeles, CA) following the approach outlined by Ferguson et al. (60); missing data were handled using full information maximum likelihood (61, 62). Ferguson et al. (60) conclude that a minimum sample size for LPA ranges from 300 to 500 participants, which we exceed. LPA was used to identify distinct groups or “profiles” of children in the sample, based on relations among the indicators of BSR, food approach and food avoidance. Each of the four BSR measures (e.g., inhibitory control, attention control, impulsivity, and Walk a Line Slowly) and five appetitive traits (e.g., slowness in eating, satiety responsiveness, enjoyment of food, food responsiveness, and emotional overeating) were entered into the LPA. Variables that did not differentiate between the profiles were removed from the analyses to improve model fit. Models with 1–8 profiles of children were estimated and compared to one another. Model fit was assessed using the Bayesian Information Criterion (BIC) and Akaike Information Criterion (AIC), in which lower scores are better, as well as the Lo-Mendell-Rubin Likelihood Ratio Test (LMR-LRT) and Bootstrap Likelihood Ratio Test (BLRT), in which the fit of a model is compared with the fit of a model with one fewer profiles. We also evaluated entropy, interpretability, and latent class size.

Once the preferred LPA model was determined, we tested whether the children in each profile differed by child and parental BMI, parental feeding practices, and sociodemographics, using the Bolck-Croon-Hagenaars (BCH) method, in which the probability that each child was in each profile was used as a weight to account for uncertainty in profile assignment and reduce bias in point estimates and standard errors of profile means. Models testing profile associations with child and parental BMI included covariates to adjust for child sex and age, parent age and college education, and household income.

## Results

### Sample Demographics

Children were, on average, 4.4 years old, ranging from 3 to 5 years; 48% of children were female and 86% were Non-Hispanic, white. Approximately 53% of parents reported a college education or higher. Slightly more than one-quarter of parents reported a household income of $49,999 or less, 24% reported an income between $50,000 and $75,000, and 48% reported an income exceeding $75,000.

### Intercorrelations

Bivariate relations among measures of behavioral self-regulation and appetite self-regulation are shown in Table 1. As expected, correlations among the measures of child behavioral self-regulation were moderately to strongly related. Children's attentional focusing and inhibitory control (reported and observed) indices were positively correlated, and both were inversely associated with impulsivity. Similarly, parent reports of children's appetitive traits on the CEBQ were correlated in the expected direction. Children's satiety responsiveness was positively associated with slowness in eating, and both measures were inversely associated with enjoyment of food. Children's food responsiveness was inversely associated with satiety responsiveness, and positively associated with enjoyment of food and emotional overeating. Enjoyment of food was also positively associated with emotional eating.

**Table 1:** Inter-correlations between measures of behavioral self-regulation (BSR) and appetite self-regulation (ASR).

	**1**	**2**	**3**	**4**	**5**	**6**	**7**	**8**	**9**
1. Attention control	1.00								
2. Inhibitory control (observed)	0.28^***^	1.00							
3. Inhibitory control (reported)	0.78^***^	0.25^***^	1.00						
4. Impulsivity	−0.36^***^	−0.12^***^	−0.57^***^	1.00					
5. Satiety responsiveness	0.04	0.02	0.10^**^	−0.13^***^	1.00				
6. Slowness in eating	−0.03	0.03	−0.01	−0.03	0.45^***^	1.00			
7. Enjoyment of food	−0.04	−0.11^**^	−0.08^*^	0.11^**^	−0.50^***^	−0.31^***^	1.00		
8. Food responsiveness	−0.07	−0.07	−0.06	0.03	−0.18^***^	−0.04	0.43^***^	1.00	
9. Emotional eating	−0.02	−0.06	−0.02	−0.00	0.04	0.05	0.09^*^	0.54^***^	1.00

* *p > 0.05*.

** *p > 0.01*.

*** *p > 0.001*.

Few relations between the behavioral self-regulation measures and appetitive traits reached statistical significance (as shown in Table 1). Children's satiety responsiveness was positively associated with inhibitory control (reported) and inversely correlated with impulsivity. Children's enjoyment of food was inversely associated with inhibitory control (reported and measured), and positively correlated with impulsivity. There were no relations between attentional focusing and appetitive traits.

### Behavioral Self-Regulation (BSR) and Appetitive Traits Profiles

When a series of LPA models with 1–8 profiles was estimated to identify patterns of BSR and appetitive traits, emotional overeating did not appear to differentiate the profiles. In general, parents reported very low levels of emotional overeating among their preschool children (*M* = 1.6 out of 5.0; *SD* = 0.6; range = 1.0 to 3.75). Therefore, this variable was removed and the LPA models were rerun. Fit statistics of the subsequent models are presented in Table 2. As shown, the LMR-LRT index indicated that the 2-profile model was superior to the 1-profile model and the 3-profile model was superior to the 2-profile model, but there was little improvement in model fit when additional profiles were added. Plots of BIC and AIC indicated two elbows at the 2-profile and 6-profile models, with smaller reductions in subsequent values. In contrast, the BLRT suggested that there was always a benefit of adding more profiles. Entropy was comparable across all models, suggesting high differentiation among profiles, with high likelihoods that children could be classified in a single profile. When interpretability was examined, it appeared that new and important profiles were emerging in the 3-profile and 4-profile model, but not in models with more than 4 profiles. Moreover, the number of children in subsequent profiles was becoming quite small, suggesting that some profiles would be considered rare and unlikely to replicate. Given this pattern of findings, the 4-profile model was selected as representing the best balance between parsimony and model fit, with profiles that were distinct, easy to interpret, and not rare.

**Table 2:** Model fit statistics.

**Latent** **classes**	**BIC**	**AIC**	**Convergence**	**LMR-LRT**	**BLRT**	**Entropy**	**Log likelihood**	**% of children in smallest class**
1	14,967	14,990	YES	–	–	1.00	−7467.9	100.0%
2	14,393	14,279	YES	0.000	0.000	0.81	−7114.6	38.1%*^a^*
3	14,248	14,092	YES	0.055	0.000	0.73	−7012.2	23.1%*^b^*
4	14,150	13,953	YES	0.225	0.000	0.76	−6933.8	15.7%*^c^*
5	14,087	13,848	YES	0.311	0.000	0.76	−6872.4	9.4%*^d^*
6	14,030	13,751	YES	0.214	0.000	0.77	−6814.6	9.7%*^e^*
7	14,029	13,708	YES	0.159	0.000	0.78	−6784.4	4.6%*^f^*
8	14,025	13,663	YES	0.182	0.000	0.79	−6752.8	4.0%*^g^*

a *Proportions for the 2-profile solution were as follows: 61.9 and 38.1%*.

b *Proportions for the 3-profile solution were as follows: 41.0, 36.1, and 22.9%*.

c *Proportions for the 4-profile solution were as follows: 35.2, 32.9, 16.4, and 15.5%*.

d *Proportions for the 5-profile solution were as follows: 35.0, 30.1, 12.7, 12.6, and 9.4%*.

e *Proportions for the 6-profile solution were as follows: 29.4, 26.8, 13.4, 10.4, 10.1, and 9.7%*.

f *Proportions for the 7-profile solution were as follows: 30.5, 25.8, 12.9, 10.2, 9.6, 6.2, and 4.6%*.

g *Proportions for the 8-profile solution were as follows: 30.5, 25.0, 11.7, 10.2, 7.6, 6.1, 4.7, and 4.0%*.

**Figure 1:**
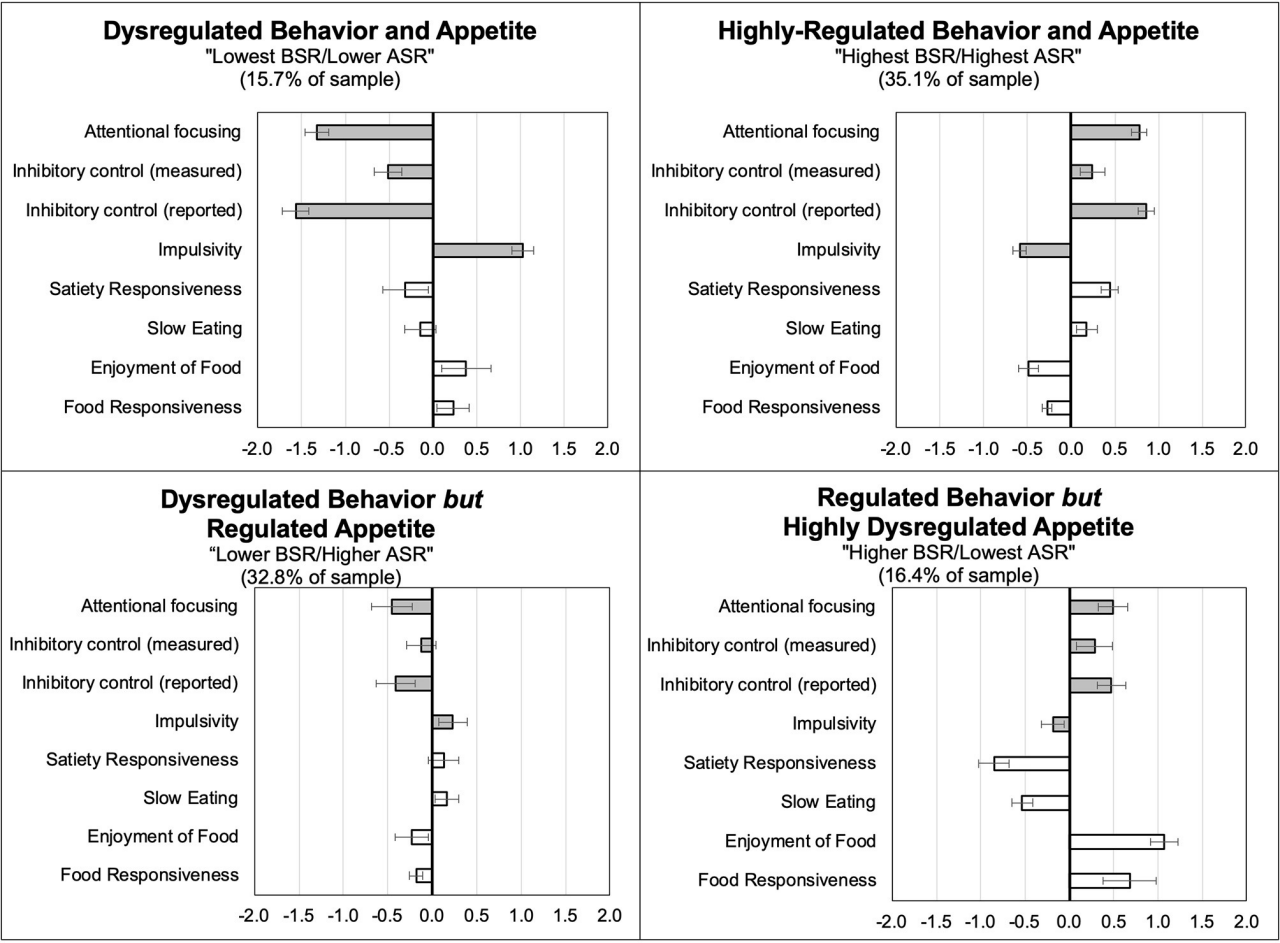
Distribution of standardized behavioral self-regulation indices (BSR; shaded bars) and appetitive traits that contribute to appetite self-regulation (ASR; white bars) by profile membership.

The four profiles were labeled based on mean differences in the BSR indices and appetitive traits; means and standard errors (SEs) are provided in Table 3 and visually depicted in Figure 1. For the sake of simplicity, we refer to food approach and food avoidance as appetite self-regulation (ASR) in the abbreviations of the profiles. As described below, two profiles displayed concordant patterns in BSR indices and appetitive traits; the remaining two profiles had discordant patterns.

**Table 3:** Distribution of standardized scores (mean ± SE) of behavioral self-regulation (BSR) and appetite self-regulation (ASR) indices by profile membership.

	**Lowest BSR/Lower ASR**	**Highest BSR/Highest ASR**	**Lower BSR/Higher ASR**	**Higher BSR/Lowest ASR**
**(Percent of sample)**	**(15.7%)**	**(35.1%)**	**(32.8%)**	**(16.4%)**
**Behavioral self-regulation (BSR)**				
Attention control	−1.3^a^	0.8^d^	−0.5^b^	0.5^c^
Inhibitory control (measured)	−0.5^a^	0.2^b^	−0.1^a^	0.3^b^
Inhibitory control (reported)	−1.6^a^	0.9^d^	−0.4^b^	0.5^c^
Impulsivity	1.0^a^	−0.6^d^	0.2^b^	−0.2^c^
**Appetite self-regulation (ASR)**				
Satiety responsiveness	−0.3^a^	0.4^d^	0.1^b^	−0.9^c^
Slowness in eating	−0.1^a^	0.2^b^	0.2^b^	−0.5^c^
Enjoyment of food	0.4^a^	−0.5^d^	−0.2^b^	1.1^c^
Food responsiveness	0.2^a^	−0.3^b^	−0.2^b^	0.7^c^

*Different superscript letters indicate significant differences between groups at p < 0.05. Higher scores on attention and inhibitory control, and lower scores on impulsivity indicate higher levels of behavioral self-regulation. Higher scores on food avoidance measures (satiety responsiveness and slowness in eating), and lower scores on food approach measures (enjoyment of food and food responsiveness) indicate higher levels of appetite self-regulation. The Lowest BSR/Lower ASR profile exhibited both dysregulated behavior and appetite; the Highest BSR/highest ASR profile exhibited both highly-regulated behavior and appetite; the Lower BSR/Higher ASR profile exhibited dysregulated behavior but regulated appetite; the Higher BSR/Lowest ASR profile exhibited regulated behavior but highly-dysregulated appetite*.

#### Concordant Profiles

- *Profile 1, Dysregulated Behavior and Appetite* (16% of the sample); children in this profile exhibited the lowest attentional focusing and inhibitory control and the highest impulsivity, as well as lower food avoidance and higher food approach (Lowest BSR/Lower ASR).
- *Profile 2, Highly-Regulated Behavior and Appetite* (35% of the sample); children in this profile exhibited the greatest attentional focusing and inhibitory control and the lowest impulsivity, along with higher food avoidance and lower food approach (Highest BSR/Highest ASR).

#### Discordant Profiles

- *Profile 3, Dysregulated Behavior but Regulated Appetite* (33% of the sample); children in this profile exhibited low attentional focusing and inhibitory control and high impulsivity, but high food avoidance and low food approach (Lower BSR/Higher ASR). Children in Profile 3 scored similarly to the Highest BSR/Highest ASR profile on two out of 4 of the ASR indices.
- *Profile 4, Regulated Behavior but Highly Dysregulated Appetite* (16% of the sample); children in this profile exhibited high attentional focusing and inhibitory control and low impulsivity, but low food avoidance and high food approach (Higher BSR/Lowest ASR). Children in Profile 4 scored similarly to the Highest BSR/Highest ASR profile on measured inhibitory control, but were the most dysregulated on all four of the ASR indices.

### Sociodemographics

Mean differences in sociodemographics across the four profiles are shown in Table 4. Both high BSR profiles had greater proportions of female and older children than the other two profiles. Children in the Highest BSR/Highest ASR profile had the highest household income levels and proportion of parents with a college education. The low ASR profiles had the lowest household incomes, and children in the Lowest BSR/Lower ASR profile were least likely to have parents with a college education.

**Table 4:** Mean (± standard error) distribution of behavioral self-regulation (BSR) and appetite self-regulation (ASR) indices by profile membership.

	**Lowest BSR/Lower ASR**	**Highest BSR/Highest ASR**	**Lower BSR/Higher ASR**	**Higher BSR/Lowest ASR**	**Total Sample**
**(Percent of sample)**	**(15.7%)**	**(35.1%)**	**(32.8%)**	**(16.4%)**	**(100.0%)**
**Demographics**					
Child sex, % female	31.4^a^	58.1^b^	40.9^a^	59.9^b^	48.1
Child age, years	4.2 ± 0.1^a^	4.5 ± 0.0^b^	4.3 ± 0.0^a^	4.6 ± 0.1^b^	4.4 ± 0.00
Parent education, % college	0.37 ± 0.1^a^	0.63 ± 0.0^c^	0.55 ± 0.0^b,c^	0.42 ± 0.0^a,b^	0.53 ± 0.0
Household income^1^	3.5 ± 0.2^a^	4.3 ± 0.1^c^	4.0 ± 0.1^b^	3.6 ± 0.2^a^	4.0 ± 0.5
**Child weight status**					
BMI^2^	17.0 ± 0.2^a^	16.1 ± 0.1^b^	16.2 ± 0.1^b^	16.8 ± 0.2^a^	16.4 ± 0.1
BMI z-scores^2^	0.9 ± 0.1^a^	0.4 ± 0.1^b^	0.4 ± 0.1^b^	0.8 ± 0.1^a^	0.6 ± 0.0
BMI percentile^2^	73.2 ± 2.6^a^	62.7 ± 2.0^b^	63.2 ± 2.2^b^	69.9 ± 2.9^a^	65.8 ± 1.0
**Food parenting practices**					
Modeling	3.9 ± 0.1^a^	4.2 ± 0.1^a,b^	4.2 ± 0.1^a,b^	4.3 ± 0.1^b^	4.2 ± 0.0
Monitoring	4.1 ± 0.1	4.0 ± 0.1	4.1 ± 0.1	4.2 ± 0.1	4.1 ± 0.0
Food as reward	2.5 ± 0.1	2.5 ± 0.2	2.6 ± 0.1	2.5 ± 0.1	2.5 ± 0.0
Emotion regulation	1.7 ± 0.1	1.7 ± 0.1	1.6 ± 0.0	1.7 ± 0.1	1.6 ± 0.0
Pressure to eat	3.1 ± 0.1^a,b^	3.0 ± 0.1^b^	3.2 ± 0.1^a^	3.3 ± 0.1^a^	3.2 ± 0.0
Restriction for health	3.1 ± 0.1	3.3 ± 0.1	3.4 ± 0.1	3.4 ± 0.1	3.3 ± 0.0
Restriction for weight	1.7 ± 0.1	1.7 ± 0.0	1.7 ± 0.0	1.7 ± 0.1	1.7 ± 0.0
Child control	2.6 ± 0.1^a,b^	2.7 ± 0.1^b^	2.6 ± 0.1^a,b^	2.4 ± 0.1^a^	2.6 ± 0.0
**Parental weight status**					
Parent BMI	29.4 ± 0.8^a^	27.9 ± 0.5^b^	28.8 ± 0.6^a,b^	27.3 ± 0.9^b^	28.4 ± 0.2

*BMI, body-mass-index; BSR, behavioral dysregulation; ASR, appetite self-regulation. The Lowest BSR/Lower ASR profile exhibited both dysregulated behavior and appetite; the Highest BSR/highest ASR profile exhibited both highly-regulated behavior and appetite; the Lower BSR/Higher ASR profile exhibited dysregulated behavior but regulated appetite; the Higher BSR/Lowest ASR profile exhibited regulated behavior but highly-dysregulated appetite*.

*Different superscript letters indicate significant differences between groups at p < 0.05*.

1 *Reported as 1 = “ < $20,000”, 2 = “$20,000 to 34,999”, 3 = “$35,000 to 49,999”, 4 = “$50,000 to 75,000”, 5 = over $75,000*.

2 *Adjusted by child age (years), sex (1 = female), and race (1 = white), household income (1 = “ < $20,000”, 2 = “$20,000 to 34,999”, 3 = “$35,000 to 49,999”, 4 = “$50,000 to 75,000”, 5 = over $75,000), parent education (1 = 4-year college completed), and parent age (years)*.

### Child BMI

Mean differences in child and parent BMI indices are shown in Table 4. Children in the two low ASR profiles had the highest (and similar) BMI indices, compared to children in the two high ASR profiles, who had similar, low BMI index scores. No other associations with child BMI indices were found.

### Parent BMI and Food Parenting Practices

Mean differences in parent BMI and food parenting practices are also shown in Table 4. The highest parent BMI was observed among children in the Lowest BSR/Lower ASR profile; this profile also had parents who reported the lowest levels of modeling healthy eating with their child. On average, children in the two high BSR profiles had parents with the lowest BMI scores, but who diverged in their reported food parenting practices. Specifically, children in the Highest BSR/Highest ASR profile had parents who reported the highest levels of child control—i.e., a measure of autonomy-granting in feeding, and lowest pressure to eat. In contrast, children in the Higher BSR/Lowest ASR profile had parents who reported using the lowest levels of child control, and the highest levels of parental modeling and pressure to eat. Lastly, children in the Lower BSR/Higher ASR had parents who reported high pressure to eat, a score that was similar to the Higher BSR/Lowest ASR profile. There were no other significant associations with food parenting practices.

## Discussion

Children's appetitive traits tap into food approach and food avoidance behaviors that are related to behavioral inhibition and approach constructs in general domains of development. General behavioral inhibition and approach behaviors have been shown to be related to biological dysregulation (63) and dysregulated eating behaviors (22, 64) in preschool children, suggesting that important processes underlying self-regulation may be common across food and Non-food-related domains. We sought to examine patterns of relations between preschool children's self-regulation in general developmental domains (e.g., inhibitory control and impulsivity) and appetitive traits related to self-regulation in the eating domain. The results from the present study confirm evidence of a clustering of regulatory behaviors across behavioral and eating domains, although the patterns did not provide clear evidence of a dichotomy (e.g., dysregulated vs. regulated). In addition to profiles of children who were either higher or lower in both BSR and appetitive traits related to ASR, we identified profiles of children with lower BSR who exhibited lower food approach and higher food avoidance, and vice versa. Profiles with children who were regulated in only one domain were not rare; ~33% of children in the sample showed dysregulated behavior but lower food approach and higher food avoidance patterns, and 16% of children in the sample showed the opposite pattern. Along with findings from other studies with preschool children (23, 34, 65), our findings provide only partial evidence for a commonality in regulation across domains of development.

Within each BSR and appetitive traits construct, there was evidence of a consistent (and expected) pattern of domain-specific self-regulation that emerged among indicators. On BSR indicators, children were either high on attention control and inhibitory control and low on impulsivity, or they were low on attention control and inhibitory control and high on impulsivity. On appetitive traits, children were either high on food approach and low on food avoidance, or they were low on food approach and high on food avoidance. This indicates that there was a reliable level of regulation or dysregulation within each self-regulation construct, highlighting the contribution of this study's findings to our understanding of the domain specificity of self-regulation, and the potential ways in which variations in BSR-appetitive trait patterning may confer risk for obesity in young children. The finding that both concordant and discordant profiles confer varying levels of risk for obesity lends support to the need for more research that examines the interplay between bottom-up and top-down regulatory processes that are implicated in the development of obesity (17).

We found partial support for our hypothesis that children who exhibited the greatest degree of dysregulation–characterized by lower BSR, higher food approach and lower food avoidance–would have the highest BMIz. Children in profiles with higher food approach and lower food avoidance had the highest BMIz, however, these profiles varied in BSR. That is, BMIz of children in the profile characterized by lower BSR, higher food approach and lower food avoidance were not significantly higher than those of children in the profile characterized by higher BSR, higher food approach and lower food avoidance. Regardless of BSR levels, children higher in food approach and lower in food avoidance had significantly lower BMIz than children in profiles characterized by lower food approach and higher food avoidance. That is, appetite-related appetitive traits appeared to be a stronger factor in uncovering individual differences in child BMIz than BSR. This suggests that eating-related regulation may be a more potent correlate of children's weight status than behavioral self-regulation, which may have implications for obesity resilience downstream. In similar findings, Rhee et al. (26) found that low-income preschool children's BMI percentiles were higher among children with lower executive function skills, who also exhibited high food responsiveness (i.e., high food approach) and low satiety responsiveness (i.e., low food avoidance). In our sample of preschoolers, higher levels of behavioral self-regulation did not appear to add additional protection against obesity risk in children with higher food approach and lower food avoidance. Tan and Holub (24) found that parent reports of 3- to 9-year-old children's eating regulation and weight status were related, but inhibitory control was not related to children's weight status. They recommended that interventions focus on eating-related, self-regulation training. Our findings suggest that appetitive traits may be an important target for obesity prevention, given that even among children with poor BSR, those with lower food approach and higher food avoidance had lower BMIz. Additional research is needed to better understand the mechanisms by which these relations exist, including links with objective measures of eating behaviors and dietary patterns, and other obesity correlates.

We also hypothesized that parent factors known to increase children's obesity risk, including parental BMI, would be higher in children who exhibited lower behavioral and appetite self-regulation. The highest parental BMIs were evident in parents of children in the most dysregulated profile, characterized by both lower BSR, higher food approach and lower food avoidance. Children in the profiles characterized by high BSR had parents with the lowest BMIs. This pattern differs slightly from the BMI findings for children: children's BMIz was lowest among children in the profiles characterized by higher ASR (lower food approach and higher food avoidance). This suggests that parental weight status may influence a number of unmeasured family/household environmental factors (*e.g*., dietary patterns, activity and sleep patterns, parents' eating style) that may be related to deficits in self-regulation. Additional research is needed to better understand these potential factors. Furthermore, obesity prevention and treatment programs may need to be tailored based on children's risk due to parental weight status.

Our findings also revealed different patterns of relations with food parenting practices by profile. There is extensive evidence confirming relations between food parenting practices, children's dysregulated eating behaviors (55) and obesity risk (35). Responsive food parenting practices, including lower levels of coercive feeding (pressure to eat) and higher levels of respect for children's autonomy in feeding (child control), were associated with the most highly-regulated profile, characterized by higher BSR, lower food approach and higher food avoidance. The lowest levels of parents' modeling of healthy eating were reported by parents of children in the most dysregulated profile, characterized by lower BSR, higher food approach and lower food avoidance. Coercive food parenting practices, coupled with children's poor inhibitory control, have been shown to have a compound effect on children's dysregulated eating behavior (37, 38). Programs focused on improving food parenting practices may hold promise for improving children's eating behaviors, which may reduce future obesity risk.

We found a greater proportion of girls and older children in the 2 profiles characterized by high BSR, with varying levels of food approach and avoidance. These findings align with those showing evidence of developmental, age-related increases in BSR, with reports of higher proficiency in girls compared to boys (66, 67). In addition, girls have been shown to follow developmental trajectories characterized by attainment of self-regulatory proficiency at younger ages compared to boys (68, 69). There is also a growing body of literature that shows evidence of sex differences in young children's appetitive behaviors. Studies described in a review by Keller et al. (70) show that the relation between appetitive traits and weight status varies by sex, with a stronger association in girls. In contrast, several studies show that self-regulation of eating (71, 72), as well as the relation between BSR and obesity risk (73, 74) varies by sex; relations appear to be more pronounced in boys. The analyses in the current study did not test whether associations between the profiles and children's BMI varied by sex. There is a need for studies that examine the combined influence of BSR and appetitive traits on children's obesity risk, and how these relations may vary by sex.

Unlike variable-centered statistical modeling approaches (e.g., multiple regression) that provide information on patterns of relations among variables, LPA allowed us to identify profiles that best represent subgroups of preschool children with similar patterns of relations among BSR indicators and appetitive traits. In fact, an examination of correlations between individual BSR indicators and appetitive traits in our sample revealed very few significant associations between individual measures of BSR and individual appetitive traits. LPA yielded unique groups of children based on similarities in their varying levels of BSR and appetitive traits, and membership in these groups was differentially associated with BMIz and obesity risk factors. Few studies have used person-centered approaches to examine BSR-ASR relations. More research is needed to better understand the way in which top-down and bottom-up regulatory processes interact and coact to form self-regulation phenotypes associated with the development of obesity. In a review of food and Non-food self-regulation, Russell and Russell (18) conclude that there are “important parallels” between general self-regulation and appetite regulation, but that they also “involve unique components and processes.” Person-centered approaches are useful tools for unpacking these common and unique components and processes, and is an area ripe for inquiry.

### Study Strengths and Limitations

This study has notable strengths. It included a large sample of preschool children, and the majority of participating childcare centers served predominantly low-income families. It collected teacher reports, parent reports, direct testing, and biometric measures to assess constructs. And, it relied on latent profile analysis to model the interplay of those constructs. For studies of complex developmental phenomena and health outcomes, such as eating behavior and obesity, person-centered approaches can answer questions about how risk and protective factors are jointly associated with outcomes. In addition, the ability to add covariates to the models affords researchers the ability to further characterize individuals in each profile, which may provide information on potential targets for prevention and treatment. These approaches will expand the literature on the interplay of general self-regulation and appetite self-regulation processes, and may provide useful information on underlying factors linking these constructs (see the paper by Russell, Leech and Russell in this special section).

However, this study is not without limitations. First, although the sample is fairly large, there was evidence of response bias; just over 50% of childcare centers included in the study served a majority of low-income families, however, the response rate for parent surveys was low (52%), and parents who completed surveys were, on average, highly educated (53% college-educated) and from higher income households. The findings may have differed (including the proportion of children identified in the various profiles) if a greater proportion of parents completed surveys. Furthermore, parent reports are subject to bias, particularly for reports related to parenting and demographics. In addition, parents' height and weight were self-reported; this may explain the trends in associations between parental BMI and the profiles. We also did not include an objective measure of eating regulation or energy intake. We are also limited in our ability to make inferences about causation or bidirectionality, given the cross-sectional study design. The racially, ethnically and socioeconomically homogenous sample limits us to generalizing the findings to preschool children in predominantly rural, Northeastern U.S. settings. In addition, we only modeled patterns of self-regulation across two distinct domains of development: behavior- and appetite-related domains. There remains a need to examine self-regulation across multiple domains of development (e.g., biological, behavioral, emotional and appetitive). There are also likely a number of potential confounding variables that were not measured in this study. Lastly, there is clear value in the use of LPA. However, there is usually some judgment involved in determining the number of subgroups and describing the characteristics of those subgroups. Confidence in the existence of particular groups will be enhanced once replicated in further studies.

### Summary and Implications

The findings from this study provide evidence of differing combinations of self-regulation across behavioral and eating domains, and the potential influence on children's obesity risk varies across self-regulation profiles. Obesity tracks from childhood through adolescence (75) and adulthood, (76) and based on the current prevalence of obesity in U.S. children, it is estimated that nearly 60% of children with obesity will become adults with obesity (77). The development of obesity in children and adolescents is particularly troubling given its links with a multitude of negative physical health outcomes, (78, 79) and psychosocial and behavioral challenges (80). Early intervention and prevention efforts should focus on improving children's regulation across developmental domains. In a review of studies linking general self-regulation to appetite-related regulation in children, Russell and Russell (17) conclude that general self-regulation increases as children age, while appetitive self-regulation appears to decrease with age. If general self-regulation is malleable, and is thought to drive regulatory behaviors in other domains of development, there is a pressing need to intervene during early childhood (2–5 years), a sensitive period when general self-regulation is rapidly developing (11, 14). Appetitive traits appear to change with age as well, with a shift toward more dysregulated traits (decreased satiety responsiveness and increased food responsiveness) as children age (28, 31). Furthermore, although there is a high degree of heritability in appetitive traits related to eating regulation, the behaviors associated with these traits are thought to be malleable (33, 81).

Findings from promising behavioral interventions and observational studies provide evidence that programs designed to improve self-regulation skills in general behavioral domains may play a role in decreasing adiposity, (82) as well as improve children's appetitive behaviors (83, 84). There is also evidence that preschool children can be taught to regulate their food intake, and focus attention on cues that signal hunger and fullness (71, 85). Lastly, interventions focused on improving general parenting and food parenting practices can impact children's behavioral self-regulation and eating behaviors in ways that reduce obesity risk (86–88). Taken together, there is evidence that behavioral and eating-related regulation factors are malleable targets for prevention and early intervention. However, our findings suggest that programs designed to improve regulation in these domains may need to be targeted based on differing patterns of children's self-regulation across developmental domains.

## Data Availability Statement

The original contributions presented in the study are included in the article/supplementary materials, further inquiries can be directed to the corresponding author/s.

## Ethics Statement

The studies involving human participants were reviewed and approved by Penn State University Office for Research Protections. Written informed consent to participate in this study was obtained from the individual(s), and minor(s)' legal guardian/next of kin, for the publication of any potentially identifiable images or data included in this article.

## Author Contributions

LF and BR conceptualized and designed the study, drafted the initial manuscript, and reviewed and revised the manuscript. BR conducted all analyses. LF provided critical oversight for the development of the analytical approach. KK, RN, and JS contributed to the interpretation of the results and preparation of the manuscript. All authors contributed to the article and approved the submitted version.

## Funding

This material is based upon work that was supported by the National Institute of Food and Agriculture, U.S. Department of Agriculture, under award number 2015-68001-23233.

## Conflict of Interest

The authors declare that the research was conducted in the absence of any commercial or financial relationships that could be construed as a potential conflict of interest.

## Publisher's Note

All claims expressed in this article are solely those of the authors and do not necessarily represent those of their affiliated organizations, or those of the publisher, the editors and the reviewers. Any product that may be evaluated in this article, or claim that may be made by its manufacturer, is not guaranteed or endorsed by the publisher.
